# The relationship between script memory for everyday events and schizotypy: an investigation through a development of Japanese Situational Feature Recognition Test

**DOI:** 10.3389/fpsyt.2024.1345789

**Published:** 2024-06-25

**Authors:** Mariko Kikutani, Yuta Takiguchi, Kota Ebina, Mie Matsui

**Affiliations:** Institute of Liberal Arts and Science, Kanazawa University, Ishikawa, Japan

**Keywords:** script, memory, schizotypy, situational feature recognition test, quality of life

## Abstract

**Background:**

Script memory is stored information about a sequential structure of an activity (e.g., going shopping), including what actors do, the purpose of the activity, and the likely consequences of the actor’s actions. It has been reported that script memory is impaired among schizophrenia patients. The present research investigated the relationship between schizotypal personality tendencies (schizotypy) and script memory by testing Japanese individuals.

**Method:**

First, a new test to measure the intactness of the script memory was created by asking the public to report activities they often perform and what behaviors each activity contains. The test contains 15 everyday activities, each accompanied by 15 behavior choices, either strongly associated, completely unrelated, or weakly associated with the activity. Next, undergraduate participants were presented with the test and chose appropriate behaviors for each activity (Study 1 and 2). Their extent of schizotypy was measured using Schizotypal Personality Questionnaire Brief (SPQ-B). Study 3 targeted the public of various ages. In addition to Study 2 procedure, participants reported the extent of psychological burden for performing each activity, their life satisfaction, and subjective evaluation of their memory ability.

**Results:**

All studies consistently found that the script memory performance was worse for individuals with higher schizotypy. Signal detection indices for accurate discrimination between correct and incorrect choices, which were *A’* in Study 1 and *C* in Study 2 and 3, negatively correlated with the SPQ-B scores (*r* = -0.16, -0.11, -0.17, respectively). Study 3 found that the relationship between schizotypy and memory task performance was mediated by the extent of psychological burden. When a signal detection index (*d’*) for the memory task was regressed on the SPQ-B, while the psychological burden scores being a mediator, the mediation effect was significant (*B* = 0.003, *SE* = 0.001, 95% CI [0.001, 0.005]).

**Conclusion:**

Individuals with higher schizotypy seem to associate unrelated behaviors to an event, forming script memory with irrelevant information, maybe due to the schizotypy symptom of having peculiar thoughts. The newly created test must be validated using a clinical population to expand its potential to be used in clinical research.

## 1 Introduction

Our everyday life is a series of events occurring one after another. An event is defined as a time segment at a given location with an identifiable beginning and end (1). Making breakfast, for example, is an event. People understand that it takes a certain amount of time (a time segment) in the kitchen (a given location). The purpose of the action (cooking food) as well as relevant object to the event (a toaster and a knife) are also understood. The beginning and end of the event is clear due to constraints of the time, location, purpose, and objects. Getting dressed in the bedroom is not included in making breakfast because it occurs in a different place, having different purposes, and involving different objects. In such a way, preparing dinner, going glossary shopping, and walking a dog are all regarded as everyday events. Each event consists of smaller chunks of events to form a hierarchical structure; making breakfast includes putting a slice of bread in a toaster and putting butter on the toast. In order to perform such everyday activities, we must be able to perform event perception, a process of identifying events (i.e., chunking time into event segments) and understanding what each event entails (2). Event perception requires access to a specific type of memory called script memory. Script memory is a combination of episodic memory and semantic knowledge about events (3). In other words, it is a set of knowledge about an event, including objects and people, their movements, purpose, and likely consequences. Script memory is also regarded as schematic representations specifying the hierarchical structure of events as well as the causal and temporal order of goal-directed actions (4). Accurate script memory enables one to achieve correct segmentations of events, appropriate filtration of irrelevant information, correct understanding of the actor’s intentions and goals, and correct prediction of event outcomes. Event perception is regarded as social cognition because the majority of events in everyday life involve interpersonal interactions (2). The present research focuses on the script memory of people with mental illnesses, especially those with schizophrenia tendency.

It has been reported that event perception is impaired among populations with mental illness, such as autism spectrum disorder (5, 6), Alzheimer’s disease (7), and schizophrenia (8, 9). Patients with these disorders sometimes forget or misrecognize the order of actions within an event and skip some necessary steps to achieve their goals.

There is evidence that schizophrenia patients have difficulty recognizing individual actions within an event. For example, patients cannot correctly order a series of actions included in an event (10). Also, they are not good at estimating the probability of a particular action occurring in an event (9). These findings suggest that schizophrenia patients do not possess accurate (or realistic) script memory. Some researchers argue against this account and suggest that patients’ script memory is likely intact, but they cannot utilize script knowledge to perceive the real world efficiently (11). The evidence for this account is that schizophrenia patients can identify individual actions within one event relatively accurately (e.g., distinguishing taking toast out of the toaster from putting butter on the toast in the event of making breakfast). At the same time, they struggle to separate multiple events with very distinctive features, such as making breakfast and going shopping (8). The evidence implies that schizophrenia patients’ script memory for each event is somewhat intact, but they cannot smoothly associate information around them with script memory in real time, where multiple events are happening sequentially.

Although the argument is still ongoing, it appears that the script memory of schizophrenia patients is less intact compared to that of healthy individuals. Corrigan and his colleagues (12–14) investigated script memory of schizophrenia patients and created an instrument called the Situational Feature Recognition Test (SFRT). This test consists of some events (e.g., reading in a library) and lists fourteen actions that are either related to the event (e.g., putting on eyeglasses; marking the page) or unrelated to it (e.g., changing a tire; eating a meal). An initial version of the test, SFRT-1, consisted of four events that are familiar to ordinary people (12, 13), and this was expanded to include nine events in the newer version, SFRT-2 (14). Five of the nine events are familiar, and the rest are unfamiliar to ordinary Americans. Each event is accompanied by fourteen action choices, among which six are associated with the events, thus correct answers. Corrigan and his colleagues compared the test performance between schizophrenia patients and normal control, using SFRT-1 (12, 13). They found that the patients selected correct responses less frequently than the control, indicated by a lower hit rate for the patients. At the same time, the patients wrongly selected incorrect actions more often than the control, as indicated by the higher false alarm rate. This tendency was also observed in a signal detection index of *A’* (see the result section for details). A higher *A’* value means better discrimination ability of correct and incorrect responses, and the patient group showed lower *A’*s compared to the control group. Recently, a Spanish version of SFRT-2 has been developed, and the previous findings were replicated with it (15, 16).

Those previous studies on the script memory of schizophrenia patients are very significant. However, this research field is still at its early stage, and the details of memory impairment phenomenon as well as its causal mechanisms are not yet clear. Regarding schizophrenia symptoms, they are often categorized into three types: “positive symptoms including hallucinations and delusion, disorganized symptoms including thought disorder and bizarre behavior; and negative symptoms including alogia, apathy, and amotivation (17, pp. 317)”. Script memory research has not yet examined the detailed relationship between the memory impairment and those symptoms.

In terms of brain mechanism, at least to our knowledge, there is no brain imaging study focusing on script memory impairment among schizophrenia patients. However, there is rich evidence for structural and functional brain abnormalities related to social cognition of schizophrenia patients (18). For example, volumes of amygdala, which is involved in emotion recognition, is smaller for patients than for healthy controls (19). During theory of mind (ToM) tasks, patients tend to show aberrant brain activities in a wide range of brain regions, including medial prefrontal cortex, premotor cortex, and medial occipitoparietal cortex (20). Furthermore, a resting-state brain activities among schizophrenia patients show negative correlations between ToM-based front-subcallosal functional connectivity and ToM performance (21). Given that script memory is one of the social cognitive domains, impairments in script memory may be associated with abnormalities in brain structures and/or functions. As described here, the script memory research on schizophrenia can set numerous research questions, and the present research focused on the SFRT as a measuring tool of script memory.

The performance of SFRT by schizophrenia patients suggests that their difficulties in event perception are caused by impaired script memory. The present research aimed to continue investigating the relationship between schizophrenic tendency and script memory. As a first step of the research, a Japanese version of SFRT was created. Instead of translating the English version, a completely new test was made because the activities included in the original version may not be familiar to the Japanese public. Also, the original is almost 25 years old, so some of the actions may have been replaced by new ones due to, for example, technological advancement (e.g., cash payment is replaced by cashless payment for shopping). In the test creation process, approximately 300 Japanese general public reported daily activities they often perform, and fifteen frequently reported ones were selected. Next, another set of Japanese participants were presented with those activities and asked to state actions or behaviors involved in them. Finally, the authors determined correct and incorrect behavior choices for each of the 15 activities based on the responses.

There are two notable differences between the original SFRT and the Japanese version. First, the Japanese one only includes familiar activities, while the original includes unfamiliar activities for Americans (e.g., making Igloo). Second, the Japanese version has three types of behavior choices for each activity, which are the ones strongly associated with the activity (correct choices), ones that are not associated with the activity (incorrect choices), and ones that are weakly associated with the activity (vague choices). The vague choices are new to this version, and they describe the behaviors that can be performed during the activity but are not essential. The vague choices were added to reveal the mechanism of the impaired performance of the test. Previous research found that schizophrenia patients tended to choose incorrect behaviors for responses and thus showed a higher false alarm rate than normal control (e.g., 12). However, this phenomenon does not reveal whether they are likely to include behaviors that *can* occur in the activity while being unessential or whether they tend to include behaviors that are seemingly (for normal control) unrelated to the activity. Setting the vague choices in the test will help clarify this question.

The newly created test was used in three online studies. It was necessary to gather responses from a large sample because the test needed validation, and recruiting many schizophrenia patients was too difficult. So, the present research tested the general population of Japan and measured their tendency toward schizotypal personality disorder or schizotypy. According to DSM-IV (22), schizotypal personality disorder has similar symptoms to schizophrenia, such as showing peculiar thoughts and behaviors, having odd beliefs, and being delusional, which are thought to stem from common biological and genetic mechanisms (23). Thus, some researchers believe that schizotypal personality disorder is the early stage of schizophrenia (24). Among the normal population, individuals with a high level of schizotypy show lower social cognition abilities (25, 26) and general cognitive abilities (27, 28) compared to individuals with low schizotypy.

The present research used the Schizotypal Personality Questionnaire Brief (SPQ-B: 29) to measure the extent of schizotypy among the non-clinical population. Past research, which targeted a normal population of university undergraduates and used SPQ-B or SPQ (non-brief version: 30), has revealed that individuals with high SPQ scores showed lower levels of empathy (31), theory of mind and awareness of social inference (32), as well as facial expression recognition ability (33, 34), compared to individuals with low SPQ scores. Thus, individuals with high SPQ are expected to perform worse on SFRT than those with low SPQ.

Studies 1 and 2 examined university undergraduates. The participants performed the SFRT and SPQ, and the relationship between script memory and schizotypy was evaluated. Study 3 targeted the general public of various ages. In addition to the two sets of questions, this study asked participants about the subjective difficulties of performing each activity, subjective evaluation of their memory ability, and quality of life (QOL, namely, life satisfaction). The study examined whether the activities with less intact script memory would relate to the increased difficulties of performing them and lower QOL. It has been reported that individuals with higher SPQ are associated with lower QOL (35). However, findings concerning the relationship between social cognition ability and QOL for individuals with high schizotypy are inconsistent. For example, Morrison et al. (36) found a positive correlation between facial expression recognition ability and QOL for people with high schizotypy tendencies. On the contrary, Brown and Cohen (33) suggest that the expression recognition ability is unrelated to QOL.

## 2 Test creation

This section describes the creation of the Japanese SFRT. The process includes three steps: 1) selecting daily activities often performed by the Japanese public, 2) determining behaviors involved in each activity, and 3) determining correct and incorrect behavior choices used in the test. The procedures 1) and 2) were taken from studies by Sakane et al. (37) and by Matsui and Arai (38). The test creation process and the procedure of Study 1 and 2 were approved by the ethical committee of the Institute of Liberal Arts and Science, Kanazawa University (No. 2020–6, approved on March 23, 2021).

### 2.1 Selecting daily activities

Three hundred twenty-one Japanese adults between 18 and 79 years of age (160 men and 161 women, mean age = 47.41, *SD* = 19.97) took part in a web survey. The survey was conducted through a research company, Macromill (https://group.macromill.com). The company distributed the online survey link to their registered members. The participants were asked to write down at least five activities they often perform in their everyday lives (for details, see 39). Some examples were shown, which were “eating a meal in a restaurant”, “shopping in a mall”, “cleaning”, and “commuting by train”. Fifteen activities, which were frequently reported and involved clearly identifiable actions, were selected for the test. They are 1) Shopping at a supermarket, 2) Cleaning home, 3) Riding a train to go somewhere, 4) Cooking, 5) Eating at a restaurant, 6) Karaoke, 7) Preparing for going out, 8) Internet shopping, 9) Getting haircut in a hairdresser, 10) Going to a hospital, 11) Having a bath, 12) Working on a computer, 13) Going for a movie, 14) Driving, 15) Taking care of pets.

### 2.2 Determining behaviors for each activity

A new group of 320 Japanese were recruited using the same method as above (147 men and 173 women, mean age = 46.08, *SD* = 14.30). Their online survey showed three activities, randomly selected from the fifteen, and asked the participants to list actions or behaviors performed during each activity. They were instructed to describe each behavior as thoroughly as possible and list them chronologically. The “taking a taxi ride” activity was presented as an example. Its related behaviors were “going to the taxi stop, raising a hand, getting in the vehicle, telling the destination to the driver, having a conversation with the driver, arriving at the destination, paying, thanking the driver, getting off the vehicle”. The participants were instructed to produce five to ten answers for each activity.

Because of the randomization, there were approximately 60 respondents for each of the 15 activities. The authors selected 5 frequently reported behaviors for each activity to use as correct choices. Those reported but not selected for the correct choices were used as incorrect choices for different activities.

### 2.3 Determining behavior choices

Initially, 17 behavior choices were determined for each activity (later reduced to fifteen). They consist of 5 correct, 5 incorrect, and 7 vague choices. The authors created those vague choices. The behavior choices included in the final form of the test are reported in **Supplementary Table S1**.

## 3 Study 1

The primary purpose of this study was to examine whether the behavior choices determined by the authors were selected (or not) as expected. The correlation between the SFRT performance and schizotypy was also analyzed.

### 3.1 Method

#### 3.1.1 Participants

Two hundred thirty-five university undergraduates (137 men and 98 women, mean age = 18.61, *SD* = 1.01) at the authors’ institution took part in the study online. They were given the study link in lectures and voluntarily participated.

#### 3.1.2 Materials

The study involved the SFRT and Schizotypal Personality Questionnaire Brief (SPQ-B). SPQ-B is the short version of the Schizotypal Personality Questionnaire created by Raine (30). The original has 74 items, and the 22-item version was created by Raine and Benishay (29). The Japanese version has been validated by Iijima et al., (40) and Ito et al. (23). Example items are “sense some person or force”, “very uneasy talking to people”, and “I am an odd, unusual person”. Participants answered each item with yes or no.

#### 3.1.3 Procedure

The SFRT and SPQ items were compiled as an online form using the Qualtrics system (https://www.qualtrics.com). At first, participants read a message about the ethical considerations of this study and answered whether they gave their consent. Those who gave the informed consent moved on to report their age and sex and then responded to the SFRT. For the SFRT, each participant was presented with randomly chosen eight activities out of fifteen. Each activity was accompanied by five correct, five incorrect, and seven vague choices, and participants were asked to select items associated with the activity. They were also instructed to rate their familiarity with each activity using a seven-point scale (1. Not at all familiar – 7. Extremely familiar). The presentation order of the activities was randomized across participants. After the SFRT, the 22 items of the SPQ-B were presented, and participants answered yes or no for each question.

### 3.2 Results and discussion

The number of choices each participant made for the eight activities was used as a dependent variable. Since each activity had five correct, five incorrect, and seven vague choices, the total number of choices was 40 for the correct and incorrect ones and 56 for the vague ones. **Table 1** reports the mean number of selected items for the three choice categories. A very high proportion of the correct choices were selected, whereas the incorrect ones were hardly selected. The vague choices were selected more often than the incorrect choices but less often than the correct ones. These trends indicate that the choices were appropriately arranged for the test.

**Table 1:** Means (SD) of the SPQ, selected number of choices, and signal detection indices in the three studies.

	Study 1	Study 2	Study 3
Correct	37.17 (4.96)	69.83 (8.68)	67.96 (7.32)
Incorrect	0.25 (0.71)	0.51 (1.11)	0.43 (2.00)
Vague	11.71 (7.50)	21.50 (13.68)	14.76 (10.42)
SPQ	8.91(4.14)	8.86 (3.98)	5.95 (4.75)
HR	93% (12%)	93% (12%)	91% (10%)
FR	1.7% (1.4%)	1.0% (1.0%)	1.1% (2.6%)
*d’*	3.86 (0.62)	4.09 (0.65)	3.89 (0.62)
*C*	0.24 (0.30)	0.31 (0.32)	0.45 (0.28)
*A’*	1.38 (0.34)	1.43 (0.41)	1.57 (1.58)
*d’2*	2.94 (0.56)	2.90 (0.50)	2.89 (0.55)
*C2*	-0.21 (0.44)	-0.29 (0.48)	-0.05 (0.42)
*A’2*	1.27 (0.24)	1.30 (0.32)	1.41 (1.39)

d’, C, and A’ were from the data without the vague choices, and d’2, C2, and A’2 were from those with the vague choices being treated as incorrect.

The SPQ contained 22 items, and those answered as “yes” were counted. The mean SPQ score is reported in **Table 1**. The internal consistency of those items in the present sample (Cronbach’s α) was 0.79, which was reasonably high and similar to the previous study (23).

The mean familiarity scores for the 15 activities are shown in **Table 2**. Most items exceeded the middle score of 4.5 and were evaluated as relatively familiar.

**Table 2:** The familiarity ratings for the 15 activities measured in Study 1 and 3.

	Study 1	Study 3
Supermarket	6.06 (1.05)	5.94 (1.33)
Cleaning	5.81 (0.90)	5.40 (1.61)
Train	5.15 (1.37)	4.13 (1.95)
Cooking	5.59 (1.22)	5.03 (1.98)
Restaurant	5.51 (1.11)	4.48 (1.71)
Karaoke	4.79 (1.60)	2.81 (1.77)
Preparation	6.24 (0.82)	5.50 (1.45)
Internet shopping	4.89 (1.44)	5.01 (1.74)
Hairdresser	5.53 (1.08)	4.61 (1.83)
Hospital	4.83 (1.32)	4.36 (1.74)
Bath	6.53 (0.71)	6.12 (1.27)
PC	6.20 (0.81)	5.48 (1.74)
Movie	5.20 (1.26)	3.52 (1.85)
Driving	3.41 (1.84)	4.51 (2.30)
Pet	3.10 (2.12)	2.69 (2.18)

The relationship between the schizotypy tendency and script memory was assessed using correlation analyses. First, the number of selections of the correct and incorrect responses was analyzed. Combining the data for the eight activities, Hit (the number of selected correct choices), Miss (the number of unselected correct choices), False alarm (FA; the number of selected incorrect choices), Correct rejection (CR; the number of unselected incorrect choices) were calculated for each participant. Then, the following signal detection indices were established ^1^: hit rate (HR), false alarm rate (FR), *d’*, *A’* and *C*. The *d’* indicates how correctly one can select correct choices and reject incorrect ones. The *C* indicates the bias between the tendency to judge any choices as correct and the tendency to judge any choices as incorrect. When there is no judgment bias between these two tendencies, *C* is close to 0. When *C* < 0, one tends to make “correct” judgments frequently, while *C* exceeding 0 means that the person is reluctant to make “correct” judgments for any choices. The *A’* is similar to *d’.* The *d’* presumes that the responses for the correct and incorrect choices were normally distributed, while the *A’* compares two samples with non-normal distribution (41). A data set derived from a relatively small number of responses (not reaching several hundred), such as the current data, is suited to be analyzed with the *A’.* Corrigan and colleagues (12, 13), who created the original SFRT, also used the *A’*.

Next, the same indices were calculated using the data with the vague choices included. This time, a selection of vague choices was counted as an error. The two versions of the *d’*, *A’*, and *C* are reported in **Table 1**.

Observing the correlations shown in **Table 3**, *A’* was significantly related to the SPQ regardless of the treatment of the vague choices, indicating that the individuals with stronger tendencies of schizotypal personality are less likely to distinguish the correct and incorrect choices accurately. Although this result was statistically significant, the correlation coefficient was small. So, another analysis was performed to confirm the existence of the relationship. The *A’* was compared between a subset of participants who showed high SPQ scores and another subset with low SPQ scores using an independent sample *t*-test. The high group consisted of approximately 10% of the participants (*N* = 33, the score ranged between 14 to 21), and the low group had a similar number of participants (*N* = 26, the score ranged between 0 to 3). The t-test was performed on those participants’ data (the vague choice excluded), and the result showed that the *A’* for the high group (*M* = 1.32, *SD* = 0.34) was significantly smaller than that for the low group (*M* = 1.53, *SD* = 0.37), *t* (57) = 2.25, *p* = .028, *d* = .59.

**Table 3:** Correlation coefficients between the SPQ scores and the SFRT response measurements in the three studies.

	Correct	Incorrect	Vague	*d’*	*C*	*A’*	*d’2*	*C2*	*A’2*
Study 1 Total	-0.06	0.09	0.08	-0.01	0.02	-0.16*	0.02	0.04	-0.15*
Inter	-0.01	-0.03	-0.04	0.01	-0.01	-0.07	0.01	0.00	-0.05
Cog	-0.08	0.11	0.15*	-0.10	0.13	-0.21**	-0.06	0.12	-0.22**
Dis	-0.07	0.13	0.10	0.10	-0.11	-0.04	0.13*	-0.07	-0.02
Study 2 Total	0.09	0.14*	0.12	0.03	-0.13*	-0.07	-0.02	-0.11*	-0.04
Inter	0.16**	0.02	0.00	0.14*	-0.15**	-0.07	0.17**	-0.10	-0.04
Cog	-0.01	0.16**	0.15*	-0.04	-0.06	-0.05	-0.15**	-0.09	-0.04
Dis	-0.01	0.15**	0.10	-0.05	-0.04	0.00	-0.09	-0.04	0.01
Study 3 Total	0.04	0.11**	0.22**	0.04	-0.15**	0.02	-0.06	-0.17**	0.03
Inter	0.05	0.05	0.08*	0.06	-0.10**	0.01	0.04	-0.09*	0.02
Cog	0.05	0.11**	0.27**	0.03	-0.16**	0.00	-0.11**	-0.20**	0.01
Dis	0.00	0.11**	0.21**	-0.01	-0.11**	0.05	-0.10**	-0.13**	0.06

Inter, Interpersonal factor; Cog, Cognitive-perceptual factor; Dis, Disorganized factor. *p < .05, **p < .01.

Schizophrenia symptoms are often categorized into three types, which are positive symptoms, negative symptoms, and disorganized symptoms, and it is argued that schizotypy symptoms also have the corresponding types (17). Rain and Benishay (29) found that the SPQ scale has a three-factor structure consisting of interpersonal, cognitive-perceptual, and disorganized factors. They are thought to correspond to the negative, positive, and disorganized symptoms of schizophrenia, respectively.

In order to examine whether the correlation pattern between the SPQ and SFRT performance differ depending on the SPQ factors, the factor-based SPQ scores were calculated for each participant and they were correlated with the SFRT indeces. The factor confirmation processes for this analysis is reported in **Supplementary Material Section 2**. **Table 3** shows that the cognitive-perceptual factor appeared to have a stronger relationship with SFRT performance (*A’* in this case) than the other two factors. It suggests that script memory impairment is associated with positive symptoms of schizophrenia more strongly than with other types of symptoms. However, the SFRT in this study had several limitations as described below, so the finding is inconclusive.

The present study suggests that the script memory for familiar activities is somewhat less accurate among people with high schizotypy. At the same time, the study revealed that the vague items of the SFRT need refining. The selection rates of the vague items varied a lot (0% to 81%). Also, some activities had many vague items with higher selection rates, while some had none. It is ideal for each activity to have vague items with higher and lower selection rates. So, this issue was addressed in the following study. Another shortcoming of the present study is that the participants only answered 8 activities among the 15 to diminish the data reliability. Therefore, the participants in the next study were presented with all the activities.

## 4 Study 2

### 4.1 Method

#### 4.1.1 Participants

Three hundred and three university undergraduates (131 men and 172 women, mean age = 19.07, SD = 1.06) participated in the study.

#### 4.1.2 Materials

From the vague choices used in Study 1, those with very high (above 79%) or low (below 10%) selection rates were removed. Study 1 used 7 vague choices for each activity, but it was reduced to 5. The vague choices were distributed to all the activities so that the variation of the selection rates was equated across all the activities as much as possible.

#### 4.1.3 Procedure

The study procedure was identical to Study 1 except that each participant responded to 15 activities and had no familiarity evaluation.

### 4.2 Results and discussion

The data was treated similarly to Study 1, and the results are shown in **Tables 1**, **3**. Each activity had 5 correct, 5 incorrect, and 5 vague choices this time, and participants responded to 15 activities. Thus, the maximum number of selected choices was 75 for each of the three choice categories. The SPQ scores for this study were similar to Study 1, and Cronbach’s α was 0.73.

The result of the correlation analysis showed that the SPQ had a relationship with the number of incorrect choice selections and *C*. It clearly indicates that the participants with a higher level of schizotypy chose a noticeable number of incorrect behaviors. The negative correlation between SPQ and *C* reflects that the response bias of giving “correct” judgments to any choices is stronger for individuals with higher SPQ than those with lower SPQ. It suggests that individuals with high schizotypy found a connection between an activity and a behavior that seems unusual to those with lower schizotypy. Similar to *A’* in Study 1, the correlation coefficient was small albeit statistically significant. Therefore, interpretation requires caution, and it is discussed in the general discussion.

When the SPQ factors were considered, the interpersonal and cognitive-perceptual factors were weakly correlated with some of the SFRT indices. However, it is hard to interpret the results due to the low coefficients.

The current result did not find clear evidence that participants with the stronger schizotypy chose more vague items than low schizotypy individuals. Considering a negative correlation between SPQ-B and incorrect choice selections, there might be a trend that high schizotypy participants chose more vague items with lower selection rates than higher ones. In order to confirm this hypothesis, the participants’ vague choice selection was further analyzed. A total of 75 vague items were divided into three groups (low, middle, and high) based on the selection rates in the present study. The low group included 28 items with 0 to 19% selection rates. The middle group comprised 28 items with 20 to 39% selection rates. The final 18 items were included in the high group, whose selection rates ranged between 40 to 69%. There was one item with a very high selection rate (75.6%), and it was removed from the analysis. Next, the number of selected items was calculated by participants for these three groups, and they were correlated with the participants’ SPQ scores. A significant relationship was found only for the low group (*r* = .128, *p* = 0.26), showing that the individuals with a higher SPQ chose more vague items than those with lower SPQ, but only when those items are very unlikely to be associated with the target activities.

This research was conducted to reveal whether people with high schizotypy are likely to include behaviors that *can* occur in the activity whilst being unessential or they tend to include behaviors that are generally unrelated to the activity. The present result suggests the latter. For the vague items with a reasonable probability of occurrence in the activity, their selection rate was not related to the SPQ level. However, the choices clearly unrelated to the activities were more likely to be chosen by the individuals with higher SPQ than those with lower SPQ. Having peculiar thoughts is a major symptom of schizotypal personality disorder and schizophrenia, and this can lead patients to create unique associations between incidents that are seemingly unrelated to normal control. Therefore, it can be inferred that the script memory of people with schizophrenia contains lots of irrelevant information or wrongly associated concepts.

The participants of this study only consisted of university students. Thus, the next study targeted people with a broader age range to see whether the findings are replicated. Also, this study asked participants about the subjective difficulties of performing each activity, their life satisfaction, and the evaluation of their memory ability. It is examined whether the activities with less intact script memory would relate to the increased difficulties of performance and lower life satisfaction.

## 5 Study 3

### 5.1 Method

#### 5.1.1 Participants

The participants were 794 Japanese above 25 years old (404 men, 388 women, and 2 unanswered, mean age = 54.86, SD = 14.69). They were registered members of a research company, Cross Marketing (https://www.cross-m.co.jp/en/), and invited to this study through the company. In order to ensure the age variation in the sample, the author set three age groups, 25 to 45, 46 to 65, and above 66, and asked the research company to invite approximately 260 people from each of those groups. The research company reimbursed participants for participation.

#### 5.1.2 Materials and procedures

The study first informed the participants that the response was collected anonymously and that participants could terminate it anytime. After reading those ethical instructions, participants reported whether they gave their consent or not. The study ended there for those who did not give their consent. This procedure was approved by the ethical committee of the Institute of Liberal Arts and Science, Kanazawa University (No. 2023–2-2, approved on September 12, 2023).

The main part of the questionnaire began with biographical questions asking participants’ age, gender, and illness history. There were two questions about illnesses, and the first asked, “Are you currently consulting health professionals for mental illness or brain abnormality (e.g., stroke, brain hemorrhage, tumor, epilepsy, Parkinson’s disease, depression, schizophrenia, alcoholism, bipolar disorder, dementia, and other neural diseases)?” Participants selected their answers from “Yes”, “No”, and “Do not want to answer”. The next question used a similar phrase and asked about their past.

The SFRT followed the biographical questions. The activities and behavior choices in the test were identical to those used in Study 2, apart from one vague item with a very high selection rate being altered. In addition to the behavior selection, questions about familiarity and the psychological burden of performing each activity were presented. The familiarity of the activities was asked in the same way as in Study 1. The psychological burden question stated, “How do you feel about performing this activity? Please answer the extent to which the following six phrases apply to you.” The six phrases, enjoyable, preferable, proactive, bothersome, hard, and depressive, were presented below the question, and participants rated each word from 1 (not at all true) to 7 (very true). The SPQ-B then followed the SFRT.

Five items were presented at the end of the questionnaire to ask participants about their quality of life (QOL) and memory ability. The authors created three QOL questions based on the WHOQOL-BREF (42). The items were “My quality of life is high”, “My daily life is restricted because of physical pain and discomfort”, and “I am enjoying my life”. The memory ability questions asked participants’ subjective evaluation of their recent memory performance. The items were “My memory has been declining recently”, and “My thinking speed has been declining recently”. They were created by referring to the items used in the study by Gifford et al. (43), who identified self-report questionnaire items that can distinguish cognitively normal people and those with mild cognitive impairment. Participants answered those five questions using 1 (not at all true) to 7 (very true).

### 5.2 Results and discussion

#### 5.2.1 SFRT performance

For the SFRT, the maximum number of selected choices was 75 for each of the correct, incorrect, and vague choice categories. The mean number of selected choices for all participants (*N* = 794) was 79.9 (*SD* = 20.1). The large SD indicates that some participants selected a very small number of choices; indeed, 15 participants selected only one for each activity. Since it is very easy to select 5 correct choices in the current SFRT, participants who made an extremely small number of choices were judged as failing to provide appropriate data. So, participants who made fewer than 39 (Mean – 2SD) choices were removed from the following analyses. Forty-two individuals were removed, and the remaining were 752 participants (374 men, 376 women, and 2 unanswered, mean age = 55.3, *SD* = 14.60). Among those, 6.6% reported the presence of a current illness, and 8.9% reported a past illness. Those percentages were relatively small, so they were included in the analysis.

The mean number of selected items for the correct, incorrect, and vague choice categories and the mean values of the signal detection indices are reported in **Table 1**. For the psychological burden, the scores for the three positive phrases were reversed so that the higher total score indicates more burden. The extent of burden for 15 activities was totaled for each participant. The mean score of the current sample was 55.0 (*SD* = 12.3). The mean burden score for each activity is reported in **Supplementary Table S3**. There were three QOL questions, and the response for one of them was reversed. The higher score indicated a higher QOL; the mean of the summed QOL scores was 12.8 (*SD* = 3.55). Finally, the summed score for the two questions about memory performance was calculated for each participant. The scores were reversed so that the higher scores indicated better memory. The mean was 7.34 (*SD* = 3.01).

#### 5.2.2 Correlation and regression analyses

The correlation analysis examining the relationship between the SPQ and the SFRT performance showed that the higher SPQ is related to the higher number of incorrect choice selections and *C* (**Table 3**). This is consistent with the result of Study 2. In this study, the number of vague item selections was also positively correlated with the SPQ. In terms of symptom types, the cognitive-perceptual factor of the SPQ seems to be most robustly related with the SFRT performance.

In addition to these relationships, the total SPQ score was negatively correlated with familiarity ratings, QOL, and subjective evaluation of memory performance. The individuals with higher schizotypy were generally less familiar with the activities in the SFRT while having lower QOL and memory performance than those with lower schizotypy. The SPQ was positively correlated with the psychological burden score, meaning that the schizotypal tendency is associated with the increased burden to perform the SFRT activities. The correlation matrix for all the variables in Study 3 is reported in **Table 4**.

**Table 4:** The correlation matrix for all the variables in Study 3.

		1	2	3	4	5	6	7	8	9	10	11	12	13	14
1	Age	−													
2	Sex	-.036	−												
3	Illness history (current)	.081	.100**	−											
4	Illness history (past)	.083*	.055	.670***	−										
5	SPQ-B	-.319***	-.065	-.176**	-.238**	−									
6	Familiarity	-.018	.260***	.049	.038	-.082*	−								
7	Burden	-.219***	-.042	-.061	-.046	.223***	-.475***	−							
8	Memory	.000	.022	.007	.079*	-.231***	-.018	-.165***	−						
9	QOL	.218***	.108***	.125***	.141***	-.396***	.278***	-.414***	.294***	−					
10	Correct items	-.069	.118***	-.024	-.055	.044	.242***	-.099***	-.057	-.017	−				
11	Incorrect items	-.003	-.035	-.051	-.082*	.111**	-.026	.029	-.012	.000	-.298***	−			
12	Vague items	-.077*	.064	-.038	-.033	.224***	.172***	-.135***	-.080*	-.067	.361***	.212**	−		
13	*d’2*	-.106***	.088*	.017	-.009	-.062	.086*	.081*	.031	-.005	.628***	-.421**	-.321**	−	
14	*C2*	.094**	-.122***	.029	.049	-.167***	-.251***	.135***	.070	.038	-.768***	-.029	-.796***	-.179**	−
15	*A’2*	-.032	-.087*	.055	.059	.030	-.077*	.049	-.020	-.051	-.252***	-.054	-.124***	-.041	.218**

*p <.05, **p <.01, ***p <.001. Results involving sex and illness history show Spearman’s correlation coefficients, while others are Pearson’s correlation coefficients.

Next, it is examined whether the signal detection indices of the SFRT were predicted by the other measures recorded in this study. The three signal detection indices (*d’2*, *C2*, and *A’2* in **Table 1**) were used as a dependent variable, and a stepwise multiple regression analysis was performed. Vague choices were regarded as errors in the calculations of these indices. The predictor variables were age, gender (male = 1, female = 2), current illnesses (yes = 1, no = 2), past illnesses (yes = 1, no = 2), the SPQ, the total familiarity score for all the activities, the burden scores, the memory scores, and the QOL scores. Step 1 of the analysis included the age, gender, current and past illnesses, and SPQ as predictors. The rest was added in Step 2. It should be noted that two participants who failed to report their gender were removed, so the sample size was 750.

*d’2* and *C2* yielded some significant results. The regression model for *d’2* was significant at Step 1, *Adjusted R^2^* = 0.027, *F* (5, 744) = 5.16, *p* < 0.001. Significant effects were found for age, *β* = -0.136, 95% CI [-0.008, -0.002], *p* < 0.001, gender, *β* = 0.082, 95% CI [0.011, 0.167], *p* = 0.025, and SPQ, *β* = -0.106, 95% CI [-0.021, -0.003], *p* < 0.01. The model improved at Step 2 with *Adj.R^2^* = 0.043, *F change* (2, 742) = 3.94, *p for R^2^ change* (Δ*R^2^*) = 0.004. The effect of age was still significant, *β* = -0.101, 95% CI [-0.007, -0.001], *p* < 0.01, but that of gender ceased to exist. The effect of SPQ remained, *β* = -0.118, 95% CI [-0.023, -0.004], *p* < 0.01. Among the newly added predictors, familiarity and burden were both significant, *ps* < 0.01 [familiarity, *β* = 0.140, 95% CI (0.002, 0.009); burden, *β* = 0.150, 95% CI (0.003, 0.011)]. Since *d’* signifies the ability to distinguish correct and incorrect choices accurately, the effect of familiarity indicates that discrimination performance is better for the more familiar activities. The interpretation of the burden effect is that the discrimination gets better for more burdensome activities. The effect of memory and QOL were absent.

The model for *C2* showed a very similar pattern to the *d’2* model, except that the effect of age was absent. The model was significant at Step 1, showing *Adjusted R^2^* = 0.037, *F* (5, 744) = 6.75, *p* < 0.001. The effect of gender, *β* = -0.114, 95% CI [-0.155, -0.036], *p* < 0.01, and SPQ, *β* = -0.163, 95% CI [-0.021, -0.008], *p* < 0.001, were found. The model greatly improved at Step 2, *Adj.R^2^* = 0.100, *F change* (2, 742) = 14.439, *p for R^2^ change* (Δ*R^2^*) < 0.001. The effect of gender and SPQ remained [gender, *β* = -0.073, 95% CI (-0.121, -0.002), *p* = 0.044; SPQ, *β* = -0.169, 95% CI (-0.022, -0.008), *p* < 0.001]. As in the *d’2* model, the familiarity and burden were both significant predictors [familiarity, *β* = -.212, 95% CI (-0.009, -0.004), *p* < 0.001; burden, *β* = 0.111, 95% CI (0.001, 0.007), *p* = 0.01]. No other effect was found. *C* signifies the bias towards including choices into the correct group (*C* < 0) or the bias towards including choices into the incorrect group (*C* > 0). Therefore, the effect of familiarity in the present result indicates that the higher the familiarity, the smaller *C* gets, meaning that participants tended to judge more choices as correct for the more familiar activities. The effect of burden showed that fewer choices were judged correct for more burdensome activities.

Both models show that men were less accurate at SFRT than women. Also, the SFRT performance was better for the younger people, while this trend is especially robust for *d’*. The most notable finding was that the higher SPQ scores predicted lower SFRT performance when all the other variables were controlled. It is crucial evidence for the robust relationship between these two variables.

Generally, the performance was better for the more familiar activities as expected. However, the better discrimination performance indicated by *d’* was present for more burdensome activities than for easy ones, which was against the prediction. People may pay more attention to difficult tasks than easy ones, contributing to better encoding of events and better script memory. Familiarity and burden also influenced *C*. Participants tended to judge more choices as correct for the more familiar and less burdensome activities than their counterparts. People are likely to perform familiar and easy activities more frequently, increasing their chance to experience some non-essential behaviors for the activities. It might have led participants to include a wider variety of behaviors as correct.

#### 5.2.3 Indirect effects of SPQ-B on SFRT through familiarity and burden

The regression analyses revealed that SPQ-B significantly predicts *d’* and *C*. At the same time, SPQ-B was correlated with familiarity and burden, which were independently related to *d’* and *C*. These results suggest a possibility that SPQ-B not only directly influences SFRT performance but also indirectly through familiarity and burden. This possibility was tested by a mediation analysis using the PROCESS program of SPSS (model 4; see 44). In this analysis, the predictor variable was SPQ-B, the dependent variable was *d’2* or *C2* in **Table 1**, and the mediator variables were familiarity and burden scores. Age and gender were included in the model as covariates. The analysis employed 5000 times bootstrapping (e.g., 45). If the 95% confidence interval for familiarity and burden contains 0, it is taken as an absence of the mediation effect.

For *d’*, the direct effect (indicated by *B*) of SPQ-B was observed [the effect = -0.013, *SE* = 0.004, *t* = -2.96, *p* = 0.003, 95% CI (-0.022, -0.004)], as well as its indirect effect [the effect = 0.002, *SE* = 0.001, 95% CI (0.000, 0.004)]. Although the total mediation effect was significant, it appears to be driven by psychological burden [indirect effect = 0.003, *SE* = 0.001, 95% CI (0.001, 0.005)] but not by familiarity [indirect effect = -0.001, *SE* = 0.001, 95% CI (-0.003, 0.000)].

The identical pattern was revealed for *C*. The direct and indirect effects of SPQ-B on the dependent variable were found [direct effect = -0.017, *SE* = 0.003, *t* = -5.26, *p* <.001, 95% CI (-0.024, -0.011); indirect effect = 0.003, *SE* = 0.001, 95% CI (0.001, 0.005)]. The indirect effect of burden was significant [indirect effect = 0.001, *SE* =0.001, 95% CI (0.000, 0.003)] but that for familiarity was not [indirect effect = 0.001, *SE* = 0.001, 95% CI (-0.000, 0.003)].

These analyses confirm that schizotypy directly influences script memory. At the same time, psychological burden mediates the relationship between SPQ-B and SFRT performance. On the contrary, familiarity was not a significant mediator, most likely due to its weak correlation with SPQ-B.

#### 5.2.4 Assessment of a psychological model for the relationship among SPQ-B, SFRT, and QOL

**Figure 1:**
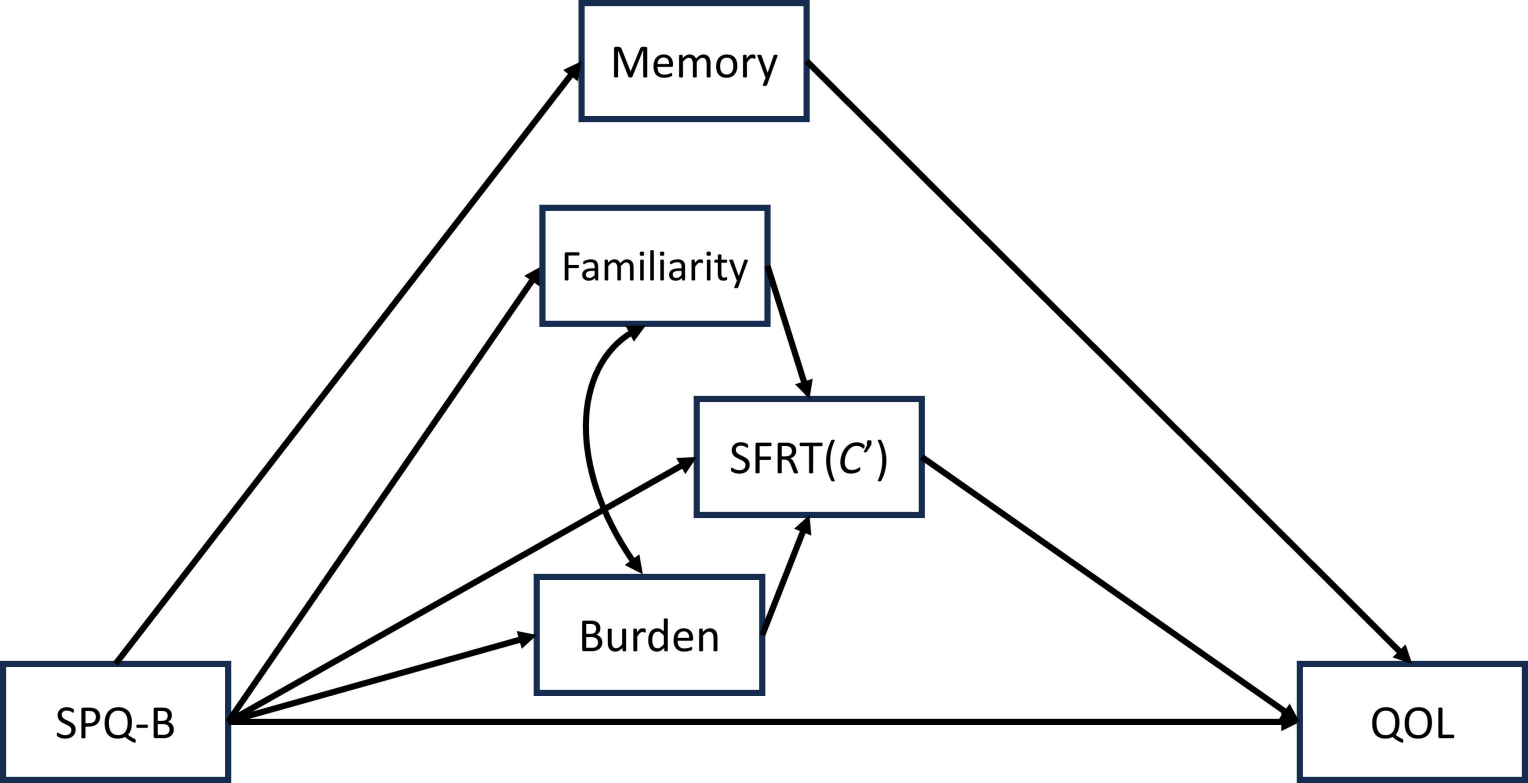
The proposed model representing the relationship among the variables in Study 3.

The QOL is thought to be the outcome of the participants’ everyday life experiences, including performing the tasks described in the SFRT. Since SFRT is revealed to be influenced by SPQ-B, familiarity, and burden, it can be hypothesized that SPQ-B works to impair script memory and consequently lower individuals’ QOL. This hypothesis can be summarized in a psychological model using all the variables in Study 3 (**Figure 1**). This section assessed the model using structural equation modeling (SEM) with the maximum likelihood estimates performed by IBM SPSS Amos 27. Age and gender were included as covariates, and covariance between familiarity and burden was assumed. The indices for the model fitness were *χ^2^*, the goodness of fit statistic (GFI), adjusted goodness of fit statistic (AGFI), comparative fit index (CFI), root mean square error of approximation (RMSEA), and Akaike information criterion index (AIC). For these indices, the following cutoffs were employed to judge the goodness of the model: *χ^2^*/*df* ≤ 5, GFI ≥. 90, AGFI ≥.90, CFI ≥. 95, RMSEA ≤.06 (46). AIC is used to compare models, and a model with a smaller AIC is the better.

**Figure 2:**
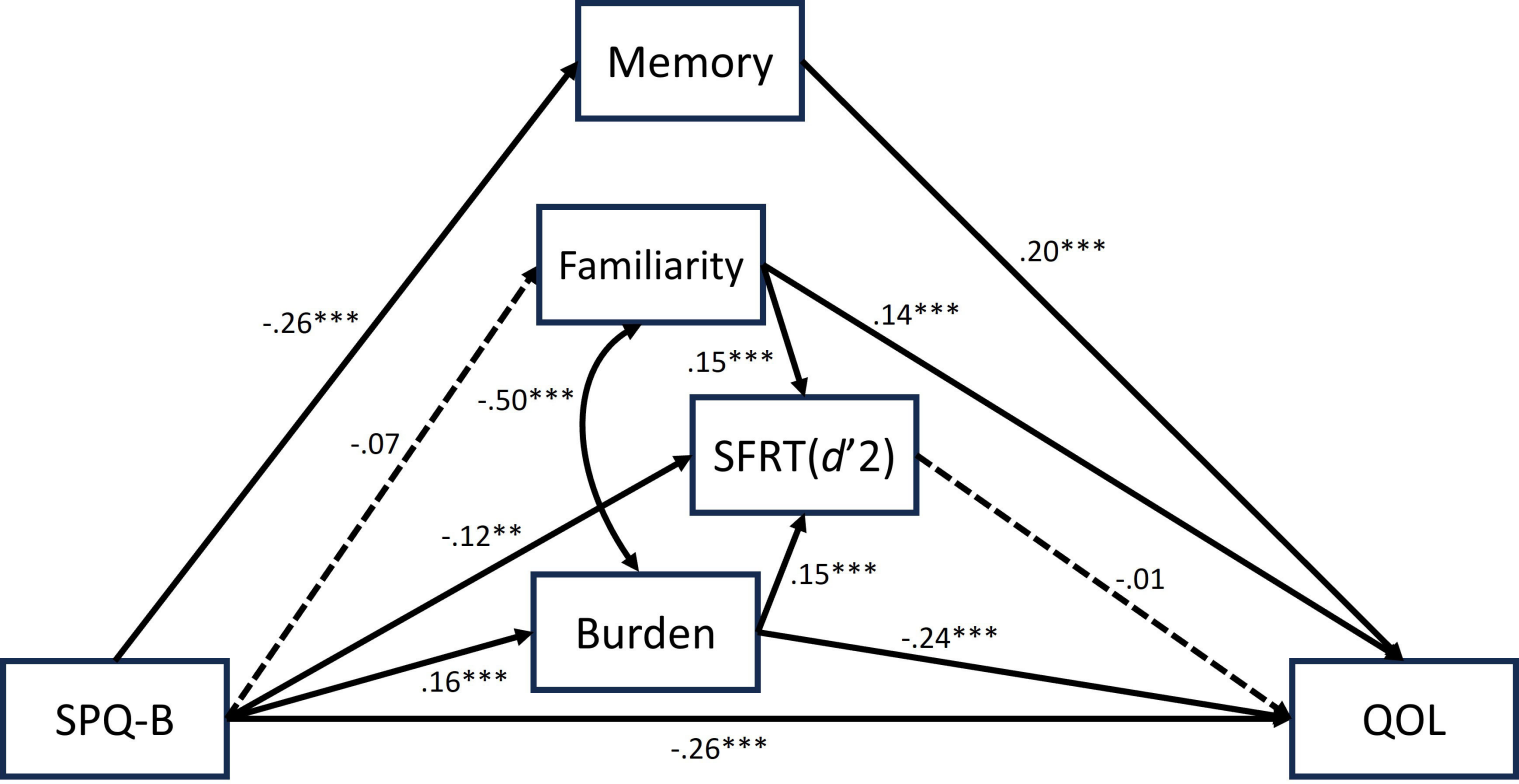
The best fit model using *d’2* for SFRT performance, with its standardized parameter estimates. Covariates (i.e., age and gender) were omitted for simplicity. The significance level of each path is indicated using the following asterisks: **p <.01, ***p <.001.

The model with *d’2* for SFRT did not meet the good fit criteria for some of the indices (*χ^2^* = 134.829, *df* = 10, *p* = .000; *χ^2^*/*df* = 13.483, GFI = .959, AGFI = .854, CFI = .838, RMSEA = .129, AIC = 186.829). The model significantly improved once paths from familiarity and burden to the QOL were added (*χ^2^* = 32.023, *df* = 8, p = .000; *χ^2^*/*df* = 4.002, GFI = .990, AGFI = .953, CFI = .969, RMSEA = .063, AIC = 88.023, see **Figure 2**).

**Figure 3:**
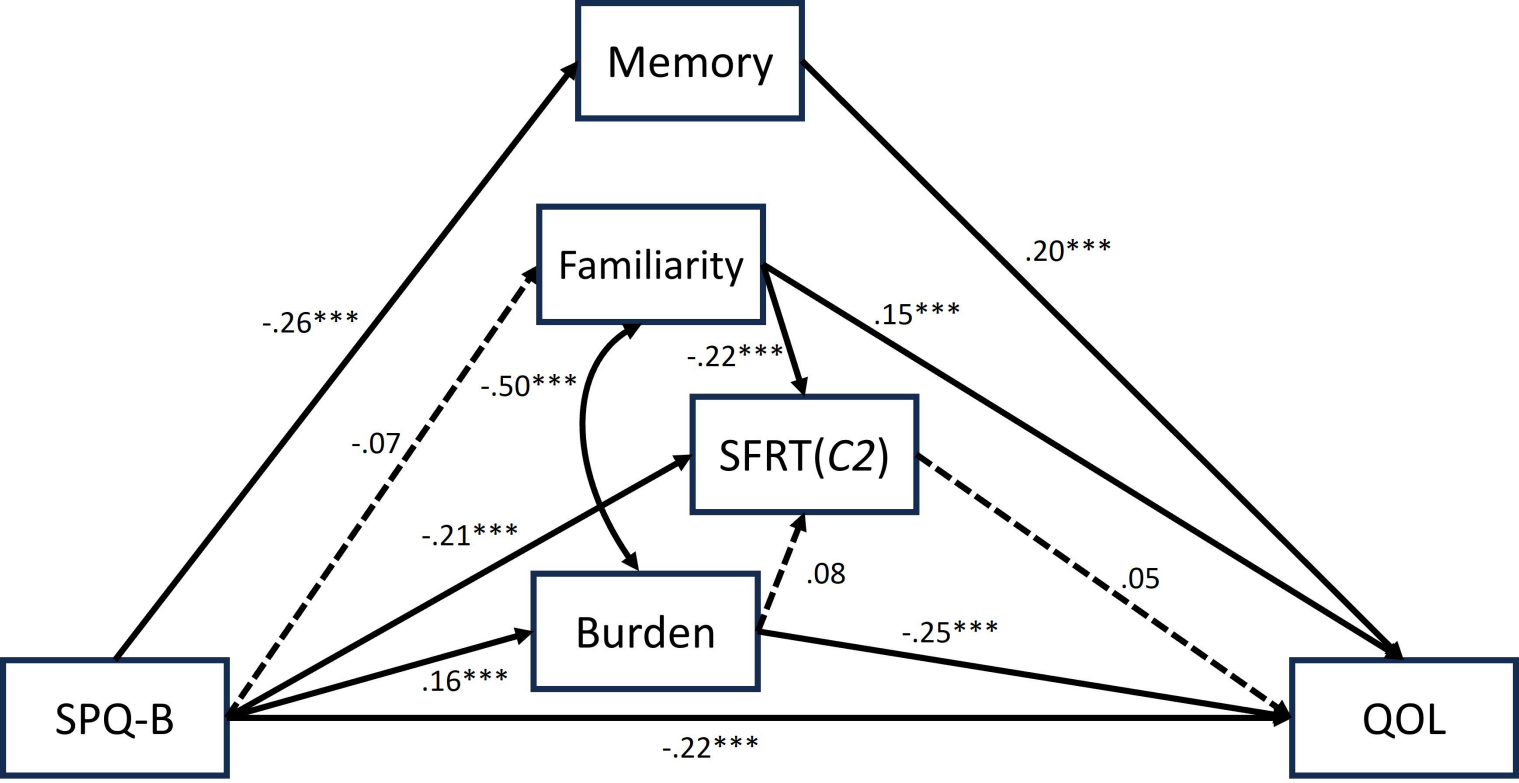
The best fit model using C'2 for SFRT performance, with its standardized parameter estimates. The estimates with three asterisks indicates that the paths are significant at *p* < .001.

The model with *C2* showed almost identical paths to the *d’2* model. The initial model was not great (*χ^2^* = 136.393, *df* = 10, *p* = .000; *χ^2^*/*df* = 13.639, GFI = .959, AGFI = .853, CFI = .846, RMSEA = .130, AIC = 188.393), but the one with additional paths was good (*χ^2^* = 31.45, *df* = 8, *p* = .000; *χ^2^*/*df* = 3.931, GFI = .990, AGFI = .954, CFI = .972, RMSEA = .063, AIC = 87.425, see **Figure 3**). One difference between the *C2* and *d’2* models was that the path from burden to *C2* was insignificant, while it was present for *d’2*.

These models revealed that schizotypy hinders SFRT performance, the subjective evaluations of memory, and QOL while increasing the activity burden. Importantly, SFRT is not directly related to QOL. Thus, individuals are unlikely to perceive impaired script memory as a cause of QOL decrease. However, burden and familiarity with SFRT activities affect QOL. Although script memory measured with SFRT is part of a memory function, SFRT performance was unrelated to subjective memory evaluation, implying that individuals do not explicitly associate these two.

#### 5.2.5 Reliability and validity of the Japanese SFRT

In this section, reliability and validity of the new SFRT are assessed using the data from Study 2 and 3. The data from Study 1 was not used because the questionnaire items had not been determined fully and participants responded to only half of the whole 15 activities. The validation process followed the study by Gómez-Gastiasoro et al. (15), which validated the Spanish version of SFRT. First, the validity was assessed by examining the internal consistency for the number of correct, incorrect, and vague responses (Correct, Incorrect, and Vague in **Table 1**). Cronbach’s alpha for correct responses were 0.94 and 0.96 in Study 2 and 3, respectively. That for incorrect responses were 0.59 and 0.87, and for vague response were 0.92 and 0.90. They were all high apart from the incorrect responses in Study 2, but once the vague choices were counted as incorrect, the values changed to 0.90 and 0.87 for Study 2 and 3, respectively. These alphas indicate that the Japanese SFRT has sufficient internal consistency.

Next, the discriminant validity, the ability of the SFRT to discriminate between high schizotypy and normal control, is assessed using Receiver Operating Characteristic (ROC) curve analysis. Study 3 data was used for this analysis. The performance of high level schizotypy participants and of the rest of the participants were compared, and discriminability was determined based on area under the curve (AUC) values (see 47, 15). The high level schizotypy in Study 3 was defined as participants whose SPQ-B score exceeded 17, based on the research by Ma et al. (48). Twenty-three participants (2.8%) were included in the high level schizotypy group. This is consistent with the proportion of high-level individuals found in Ma et al. (48) which was 2.9%.

**Figure 4:**
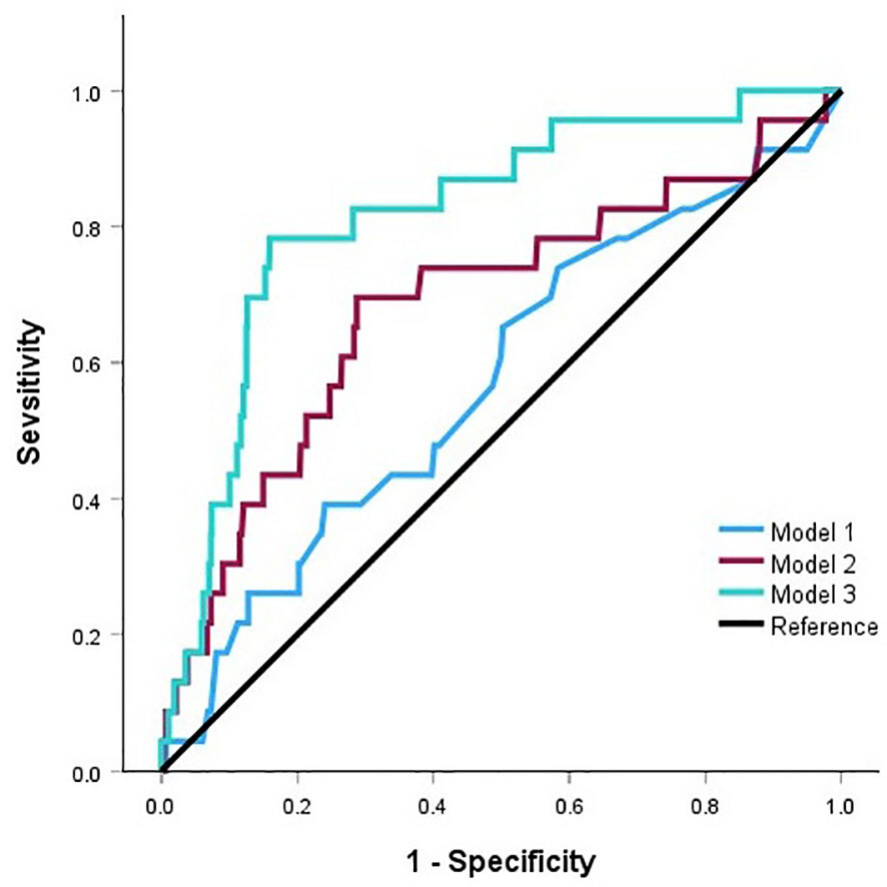
The results of the ROC analysis in Study 3.

The result of the ROC analysis is reported in **Figure 4**. Three models were created for this analysis. The first one used correct and incorrect choices but not vague choices to predict the high level of schizotypy. The AUC index of this model was 0.57 and failed to show a statistically significant difference from chance level (*p* = 0.26). The second model included vague choices into incorrect category to predict schizotypy. The AUC index of this model was 0.69 and it was significant (*p* = 0.004), suggesting that this predictive model was almost fair. Finally, participants’ age and gender were added to the second model since they might be related to schizotypy, and the participants’ age in Study 3 varied largely. The AUC index of the final model was 0.82, *p* < 0.001, with 69.6% sensitivity and 87.3% specificity. This is almost equivalent to the discriminability index of the Spanish SFRT, though the current sensitivity is lower than the Spanish version due to the unbalanced sample sizes between the high schizotypy group and the rest of the participants. Thus, it is reasonable to conclude that the Japanese SFRT can discriminate individuals with high and low schizotypy reasonably well.

## 6 General discussion

The present research investigated whether schizotypy is related to impaired script memory. The new version of SFRT was created and presented to a normal population in Japan. Schizotypy was measured using SPQ-B, and the relationship between SFRT performance and SPQ-B scores was examined. The research concludes that schizotypy affects individuals to perform very common everyday activities. As Study 3 showed, the relationship between the SFRT and SPQ remained robust when other variables were controlled.

In this research, the relationship between SFRT performance and SPQ-B was examined using correlation while previous research established the relationship by comparing the performance between the schizophrenia patients and healthy control. It was because the present research treated schizotypy as an individual variation rather than as a category. Therefore, it is impossible to compare the current and previous results directly. Also, the correlations found in the present research were relatively low (*rs* < 0.2, see **Table 3**), leaving concerns for interpretation. However, the multiple regression analyses (section 5.2.2) and discriminant validity analysis (section 5.2.4) clearly indicate that the present results sufficiently demonstrated the relationship between SFRT and SPQ-B.

One of the most significant contributions of this research to the field of psychiatry is the creation of the new SFRT. Although the test is easy to answer and has a simple format, it has sufficient sensitivity to reflect performance differences due to schizotypy among the non-clinical population. The presence of vague choices especially helps reveal slight differences in script memory performance. This test must be validated using a clinical population of schizophrenia. Once validated, the test will have a great potential to be used to measure script memory not only for schizophrenia but also for other mental illnesses. Different illnesses can result in differing performance patterns (e.g., how vague choices are selected), which should help pursue many research questions regarding patients’ memory. Accessing a large number of schizophrenia patients to validate this test will not be easy and will take time. While validation with patients is ongoing, the present research, especially Study 3, can be repeated with much larger samples (several thousands). This way, it will be possible to obtain large amount of data from people with quite high level of schizotypy, and the scope of this test for clinical population will be much more clearly indicated than in the current research.

In terms of the relationship between the SFRT and SPQ, the three studies in the present research consistently found that the individuals with higher SPQ chose more incorrect answers that are not at all associated with the target activity than those with lower SPQ. At the same time, it was not the case that individuals with high SPQ overestimated the occurrence of behaviors that are weakly related to the activities. These findings suggest that script memory for high SPQ individuals consists of many associations between an event and seemingly (for people with low SPQ) unrelated behaviors. It is likely related to one of the symptoms of schizotypal personality disorder, having peculiar thoughts. Individuals with high schizotypy may create a unique relationship between an event and behavior based on their unique thoughts. Relating to peculiar thoughts, schizotypal personality disorder patients are reported to have limited capacity to think about their mental status abstractly (49). Abstraction of one’s actions and state is a crucial part of forming script memory because individuals must extract the essence of an event from various factors. Failing to do so will likely result in script memory with lots of irrelevant information.

Previous research with schizophrenia patients reported difficulty ordering a series of actions in an event (10) and accurately estimating the probability of a particular action occurring in an event (9). Those phenomena are likely to be observed if patients have script memory with many unnatural associations of behaviors and events. It has been debated whether the patients’ script memory itself is disrupted or the memory is intact, but the process of utilizing script knowledge to perceive the real world is disrupted (11). The present research supports the former account. However, it is important to note that participants with lower SFRT performance did not report their memory decline in Study 3, meaning that impaired script memory is not overtly associated with memory problems. This may signify the unique quality of script memory, which is clearly distinct from ordinary episodic or descriptive memory.

Study 3 revealed that schizotypy affects individuals’ performance, familiarity, and feelings towards everyday activities. However, the performance of SFRT was not directly related to QOL. It is in line with the finding of Brown and Cohen (33) that the disrupted ability of facial expression recognition among high schizotypy individuals is not related to QOL. It might be the case that the crucial factor for lower QOL is not the accuracy of expression recognition but biased interpretation of expression, such as interpreting others’ expressions negatively (33) or low self-esteem due to the impaired recognition ability (36). In the present study, the psychological burden might be a crucial factor in affecting QOL rather than intactness of script memory because it was a significant mediator between SFRT and SPQ-B.

The present research not only found a crucial aspect of disrupted script memory for people with high levels of schizotypy but also created and validated a new test for script memory. However, the finding is limited to a normal population, and evaluating the test and findings with a clinical population is essential. Also, it is necessary to clearly distinguish schizotypy, schizotypal personality disorder, and schizophrenia in future research because it is not well established whether they share a common script memory deficit. In terms of the symptoms, those for schizophrenia and schizotypy are viewed as corresponding to each other. Positive, negative, and disorganized schizophrenia symptoms are thought to be equivalent to cognitive-perceptual, interpersonal, and disorganized symptoms of schizotypy, respectively (17). The Japanese SFRT performance seems to be related with cognitive-perceptual symptoms of schizotypy more strongly than with the other two symptom categories. This makes sense since the cognitive-perceptual symptoms includes peculiar thoughts and unusual ideas, which is likely behind the error in the SFRT performance caused by connecting two seemingly unrelated behaviors or events. However, the discovered correlations between SFRT performance and the SPQ factors were small, and it is impossible to distinguish individuals’ symptom types based on the SFRT performance. Future research focusing on the effect of symptom types on script memory may reveal whether the memory deficit for schizotypy and schizophrenia have a common mechanism.

Script memory is a unique type of memory and has not yet been researched thoroughly in the field of psychiatry, while it has a huge impact on people’s everyday lives. Future studies addressing shortcomings of the present research should contribute greatly to understanding the cognitive mechanisms of patients with mental illnesses.

## Data availability statement

The raw dataset used for the analyses in this manuscript can be found in the Open Science Framework repository: https://osf.io/32tvw/.

## Ethics statement

The studies involving humans were approved by The ethical committee of the Institute of Liberal Arts and Science, Kanazawa University. The studies were conducted in accordance with the local legislation and institutional requirements. The ethical consideration of the research were given to the participants at the beginning of the study, and they were asked whether they agree with participating. The agreement answer was taken as a given consent, and those who disagreed terminated the study without answering any other question.

## Author contributions

MK: Conceptualization, Data curation, Formal analysis, Investigation, Methodology, Project administration, Visualization, Writing – original draft, Writing – review & editing. YT: Data curation, Formal analysis, Visualization, Writing – original draft. KE: Data curation, Formal analysis, Visualization, Writing – original draft. MM: Conceptualization, Funding acquisition, Investigation, Methodology, Supervision, Writing – review & editing.

## Footnotes

1 The formulas for the signal detection indices used in the study were as follows.

$$\begin{array}{c} \mathrm{HR}=\;\mathrm{Hit}\;/\;(\mathrm{Hit}\;+\;Miss);\\ \;\mathrm{FR}=\;\mathrm{FA}\;/\;\left(\mathrm{FA}\;+\;\mathrm{CR}\right);\;\mathrm{in}\;\mathrm{case}\;of\;\mathrm{FA}\;=\;0,0.5\;/\;\left(\mathrm{FA}\;+\;\mathrm{CR}\right)\;\mathrm{was}\;\mathrm{used};\\ d'=\;z\left(\mathrm{HR}\right)\;–\;z\;\left(\mathrm{FR}\right);\\ C=\;-0.5\;*\;\left(z\;\left(\mathrm{HR}\right)\;+\;z\;\left(FR\right)\right);\\ A'=\;0.5\;+\;\left(\left(\mathrm{Hit}\;-\;\mathrm{FA}\right)\;\left(1+\;\mathrm{Hit}\;-\;\mathrm{FA}\right)\right)\;/\;4\;*\;\mathrm{Hit}\;\left(1\;-\;\mathrm{FA}\right) \end{array}$$
