# Supplementary material for: The relationship between script memory for everyday events and schizotypy: an investigation through a development of Japanese Situational Feature Recognition Test
Source: Front Psychiatry. 2024 Jun 25;15:1345789. doi: 10.3389/fpsyt.2024.1345789 (PMC11231421; doi:10.3389/fpsyt.2024.1345789)
Supplement: Supplementary file 1 [file Table_1.docx]

Supplementary Material

# Supplementary Table S1. The list of the SFRT choices for the 15 activities; five each for correct, incorrect, and vague category.

|  | **1. Shopping at a supermarket** |
| --- | --- |
| Correct | Choosing products, 97.0%; Queuing at cashier, 96.0%; Taking a basket or cart, 96.0%; Paying, 95.7%; Packing goods, 91.4% |
| Incorrect | Cooking rice, 6.3%; Taking clothes off and put them in the washing machine, 1.3%; Get my hair washed, 0.0%; Saving data on a USB, 0.0%; Doing press-ups, 0.0%, |
| Vague | Going to the car park, 42.9%; Looking at the smartphone, 32.3%; Wearing a hat and jacket, 22.1%; Descending stairs, 12.2%; Making a call to my family, 6.0% |
|  | **2. Cleaning home** |
| Correct | Taking rubbish out for collection, 96.7%; Hoovering, 96.4%; Dusting, 95.0%, Wiping, 92.4%; Clearing stuff on the floor, 88.8% |
| Incorrect | Entering destination information into the satnav, 0.7%; Meeting up with someone, 0.0%; Waiting in line, 0.0%, Pressing the brake pedal, 0.0%; Going for a reception, 0.0% |
| Vague | Crushing cardboard boxes, 75.6%; Folding clothes, 50.2%; Wearing a mask, 36.6%; Checking the stock of daily necessities, 25.1%; Closing the door, 11.9% |
|  | **3. Riding a train to go somewhere** |
| Correct | Getting on a train, 98.3%; Waiting at a platform, 96.4%; Purchasing a ticket, 96.4%; Going through a ticket gate, 94.7%; Checking the train schedule and platform, 92.1% |
| Incorrect | Drying hair, 3.6%; Changing tyers, 3.0%; Moving furniture, 1.0%; Ironing, 0.0%; Making salad, 0.0% |
| Vague | Consulting a map, 65.7%, Checking weather, 58.7%, Putting shoes on, 40.3%; Withdrawing cash, 30.0%; Purchasing drink, 28.1% |
|  | **4. Cooking** |
| Correct | Seasoning, 98.7%; Chopping and peeling ingredients, 97.7%, Frying, grilling, boiling, 97.0%; Putting meal on plates, 95.7%; Washing ingredients, 92.4% |
| Incorrect | Evaluating online shops, 1.3%; Hammering nails, 0.7%; Riding a bicycle, 0.0%; Holding a microphone, 0.0%; Folding clothes, 0.0% |
| Vague | Burning myself, 55.1%; Throwing rubbish away, 47.9%; Taking off accessories and watches, 30.0%; Singing, 6.3%, Watching TV, 2.6% |
|  | **5. Eating at a restaurant** |
| Correct | Eating and drinking, 98.3%; Ordering, 96.7%; Looking at the menu, 96.0%; Paying, 92.4%; Guided to the table, 90.1% |
| Incorrect | Preparing cooking utensils, 4.3%, Shampooing, 0.7%, Holding a shopping basket, 0.7%; Clicking a mouse, 0.0%; Sharpening pencils, 0.0% |
| Vague | Queuing, 59.4%; Taking photos, 32.7%; Tying hair, 11.2%; Presenting ID, 10.9%; Going to a bank, 4.3% |
|  | **6. Karaoke** |
| Correct | Ordering drink and food, 96.4%; Singing, 96.0%; Entering a booth, 91.7%; Paying, 82.2%; Choosing songs to sing and type the song number in the machine, 78.2% |
| Incorrect | Turning washing machine on, 0.0%; Warming bath up, 0.0%; Watering flowers, 0.0%; Receiving prescriptions, 0.0%, Stapling documents, 0.0% |
| Vague | Going to the toilet, 37.6%; Checking point cards, 31.4%; Turning off the light, 17.8%; Playing the stone, paper scissors, 11.6%; Turning off the mobile phone, 1.3% |
|  | **7. Preparing for going out** |
| Correct | Packing personal belongings to take and check them, 96.0%; Choosing what to wear and change, 94.1%; Setting hair, 89.1%; Washing face, 82.5%; Brushing teeth, 82.2% |
| Incorrect | Buying film pamphlet and goods, 4.0%; Filling a medical questionnaire, 1.3%; Tasting the cooking, 0.3%; Playing the guitar, 0.3%; Throwing a ball, 0.0%, |
| Vague | Checking the route, 54.8%; Contacting friends, 38.0%; Writing a shopping list, 23.4%; Watching news, 12.5%; Checking the contents of the fridge, 10.9% |
|  | **8. Internet shopping** |
| Correct | Completing checkout, 97.4%; Entering purchase information, 95.7%, Opening the website with PC or smartphone, 94.1%; Searching products, 93.7%; Checking the product price and reviews, 91.7% |
| Incorrect | Going through a ticket gate, 2.3%; Packing products in a bag, 0.3%; Putting apron on, 0.3%; Calling a shop staff, 0.0%; Polishing shoes, 0.0% |
| Vague | Sitting on a chair, 22.1%; Looking up a calendar, 16.8%; Sending an email, 16.2%; Talking with family, 12.2%; Turning a light on, 8.9% |
|  | **9. Getting haircut in a hairdresser** |
| Correct | Having a haircut, 98.3%; Requesting hairdresser the style, 97.4%; Checking the completed style, 91.7%; Paying, 81.5%; Sitting on the treatment chair, 74.9% |
| Incorrect | Turning the cooker on, 1.3%, Putting PC away, 0.7%; Putting rubbish out, 0.3%; Preparing cleaning equipment, 0.0%; Eating meal, 0.0% |
| Vague | Reading magazines, 57.1%, Taking a jacket off, 54.8%; Going to see someone, 29.0%; Wearing contact lenses, 14.2%; Getting on the bus, 11.6% |
|  | **10. Going to a hospital** |
| Correct | Being seen by a doctor, 98.3%; Entering the examination room, 97.7%, Going through a reception, 95.7%; Waiting in line, 92.7%, Paying, 76.6% |
| Incorrect | Putting products in a basket, 0.7%; Booking seats in a cinema, 0.7%; Adding bubble bath in bath water, 0.3%; Perform weight training, 0.3%; Waiting for food, 0.0% |
| Vague | Greeting, 50.5%; Wearing slippers, 50.5%; Parking the car at the carpark, 49.5%; Looking at the guide map, 37.0%; Weighing myself, 25.7% |
|  | **11. Having a bath** |
| Correct | Washing body, 96.7%; Washing hair, 94.4%; Staying in a bathtub, 92.4%; Taking clothes off, 92.1%; Wiping body, 91.7% |
| Incorrect | Paying, 3.0%; Shifting the gear, 0.0%; Sitting in front of PC, 0.0%; Opening a photo album, 0.0%; Putting a pan over the fire, 0.0% |
| Vague | Standing on a scale, 26.7%; Cleaning a drain, 24.1%; Stretching, 18.5%; Watching video clips, 8.3%; Cutting nails, 4.6% |
|  | **12. Working on a computer** |
| Correct | Starting up a software, 97.4%; Opening electronic files and web pages, 97.4%; Turning PC on, 97.0%; Turning PC off, 94.1%; Keyboard typing, 82.2% |
| Incorrect | Choosing what to wear, 0.3%; Oiling a pan, 0.0%; Showering, 0.0%; Sprinkling water with a hose, 0.0%; Cutting cake, 0.0% |
| Vague | Listening to music, 30.7%; Taking notes, 27.1%; Drinking, 17.8%; Recording a video clip, 15.2%; Using eye drops, 14.9% |
|  | **13. Going for a movie** |
| Correct | Buying tickets, 96.0%; Watching a film, 96.0%; Checking the time and room number for the film, 96.0%; Sitting down in the cinema room, 95.7%; Buying food and drink, 81.5% |
| Incorrect | Shampooing, 1.7%; Wiping floor, 0.3%; Putting cleaning equipment away, 0.0%; Telling a doctor about symptoms, 0.0%; Turning the cooker on, 0.0% |
| Vague | Talking with friend, 44.6%; Looking for discount coupons, 41.3%; Getting on a lift, 33.7%; Going to the station, 28.1%; Looking up information for close restaurants, 19.1% |
|  | **14. Driving** |
| Correct | Pressing the accelerator, 97.4%; Turning the engine on, 97.0%; Holding a steering wheel, 96.7%; Putting a seatbelt on, 96.4%; Unlocking the car door, 89.4% |
| Incorrect | Opening an umbrella, 1.7%; Looking up drink menu, 0.3%; Setting a table to dine, 0.0%; Changing shampoo bottles, 0.0%; Washing face, 0.0% |
| Vague | Clearing steamed windows, 61.1%; Turning aircon on, 46.2%, Checking belongings to take, 35.0%; Wearing sunglasses, 33.3%; Having conversations, 25.4% |
|  | **15. Taking care of pets** |
| Correct | Giving food, 96.7%; Going for walk, 94.1%; Playing together, 91.1%; Cleaning excrement, 90.1%; Brushing and clipping nails of the animal, 89.8% |
| Incorrect | Lifting dumbbells,5.6%; Typing password in, 0.7%; Copying sutra, 0.3%; Taking off the seatbelt, 0.0%; Having bath, 0.0% |
| Vague | Taking photos, 29.4%; Washing hands, 28.4%; Clapping hands, 26.1%; Traveling far, 10.2%; Tying shoelaces, 5.6%, |

# Note: The reported values are selection rates of these choices in Study 2.

# Supplementary Materials Section 2. The description of the factor confirmation process for the SPQ-B.

Raine and Benishay (1995) found three factors of the SPQ-B, which are Cognitive-perceptual, Disorganized, and Interpersonal. In the present research, a confirmatory factor analysis was performed on Study 1 data to see whether the original factor structure fit them. The result was unsatisfactory, so a new exploratory factor analysis was performed on the same data. The result revealed a three-factor structure like Raine and Benishay (1995), but questionnaire item allocations slightly differed from theirs (Supplementary Table S2-2). Further confirmatory factor analyses performed for Study 2 and Study 3 data revealed that the newly found (data-driven) factor structure fitted both data set reasonably (Supplementary Table S2-1, S2-4). Internal consistencies were also acceptable (Supplementary Table S2-3). The factor based SPQ-B scores were calculated using the new structure shown in Supplementary Table S2-4.

Supplementary Table S2-1. The results of the confirmatory factor analyses.

|  | chi-square | df | p | GFI | CFI | RMR | RMSEA | 90%CI (RMSEA) | |
| --- | --- | --- | --- | --- | --- | --- | --- | --- | --- |
| Study 1 |  |  |  |  |  |  |  |  |  |
| 3-factor (original) | 507.488 | 206 | .000 | .821 | .579 | .021 | .079 | .070 | .088 |
| 3-factor (data-driven) | 347.709 | 206 | .000 | .881 | .802 | .017 | .054 | .044 | .064 |
| Study 2 |  |  |  |  |  |  |  |  |  |
| 3-factor (data-driven) | 451.704 | 206 | .000 | .881 | .722 | .016 | .063 | .055 | .071 |
| Study 3 |  |  |  |  |  |  |  |  |  |
| 3-factor (data-driven) | 853.416 | 206 | .000 | .901 | .858 | .010 | .065 | .060 | .069 |

Supplementary Table S2-2. The factor structure of SPQ-B in Study 1.

| Item No. | Interpersonal | Disorganized | Cognitive-perceptual |
| --- | --- | --- | --- |
| 11 | .713 | -.003 | .022 |
| 16 | .643 | -.071 | -.244 |
| 21 | .642 | .113 | -.162 |
| 14 | .527 | -.109 | .223 |
| 18 | .467 | -.051 | .334 |
| 22 | .456 | -.050 | -.042 |
| 20 | .448 | .376 | .019 |
| 15 | .368 | .207 | -.070 |
| 12 | -.245 | .639 | -.334 |
| 17 | .057 | .605 | -.110 |
| 13 | -.208 | .552 | .090 |
| 9 | .082 | .504 | .184 |
| 7 | .179 | .503 | .025 |
| 2 | .069 | .423 | .132 |
| 3 | -.145 | .399 | .330 |
| 8 | .191 | .383 | .152 |
| 4 | .038 | .365 | .059 |
| 19 | -.004 | -.041 | .827 |
| 1 | -.227 | .002 | .778 |
| 6 | -.068 | .159 | .625 |
| 10 | .265 | .080 | .340 |
| 5 | .048 | .014 | .315 |

Supplementary Table S2-3. Internal consistency (Cronbach’s alpha) for the three factors in Study 1, 2, and 3.

|  | Study 1 | Study 2 | Study 3 |
| --- | --- | --- | --- |
| Interpersonal (8 item) | .695 | .689 | .797 |
| Cognitive-Perceptual (9 item) | .654 | .611 | .770 |
| Disorganized (5 item) | .623 | .565 | .755 |

Supplementary Table S2-4. SPQ-B items and their factors.

| Item No. | Raine & Benishay (1995) factor | Current factor |  |
| --- | --- | --- | --- |
| 2 | Cognitive-perceptual | Disorganized | Have you ever had the sense that some person or force is around you, even though you cannot see anyone? |
| 4 | Cognitive-perceptual | Disorganized | Are you sometimes sure that other people can tell what you are thinking? |
| 5 | Cognitive-perceptual | Cognitive-perceptual | Have you ever noticed a common event or object that seemed to be a special sign for you? |
| 9 | Cognitive-perceptual | Disorganized | Do you often pick up hidden threats or put-downs from what people say or do? |
| 10 | Cognitive-perceptual | Cognitive-perceptual | When shopping do you get the feeling that other people are taking notice of you? |
| 12 | Cognitive-perceptual | Disorganized | Have you had experiences with astrology, seeing the future, UFOs, ESP, or a sixth sense? |
| 16 | Cognitive-perceptual | Interpersonal | Do you ever suddenly feel distracted by distant sounds that you are not normally aware of? |
| 17 | Cognitive-perceptual | Disorganized | Do you often have to keep an eye out to stop people from taking advantage of you? |
| 3 | Disorganized | Disorganized | People sometimes comment on my unusual mannerisms and habits. |
| 6 | Disorganized | Cognitive-perceptual | Some people think that I am a very bizarre person. |
| 8 | Disorganized | Disorganized | Some people find me a bit vague and elusive during a conversation. |
| 13 | Disorganized | Disorganized | I sometimes use words in unusual ways. |
| 19 | Disorganized | Cognitive-perceptual | I am an odd, unusual person. |
| 20 | Disorganized | Interpersonal | I find it hard to communicate clearly what I want to say to people. |
| 1 | Interpersonal | Cognitive-perceptual | People sometimes find me aloof and distant |
| 7 | Interpersonal | Disorganized | I feel I have to be on my guard even with friends. |
| 11 | Interpersonal | Interpersonal | I feel very uncomfortable in social situations involving unfamiliar people. |
| 14 | Interpersonal | Interpersonal | Have you found that it is best not to let other people know too much about you? |
| 15 | Interpersonal | Interpersonal | I tend to keep in the background on social occasions. |
| 18 | Interpersonal | Interpersonal | Do you feel that you are unable to get "close" to people? |
| 21 | Interpersonal | Interpersonal | I feel very uneasy talking to people I do not know well. |

# Supplementary Table S3

The mean burden scores for the 15 activities.

|  | Mean | S.D |
| --- | --- | --- |
| Supermarket | 3.43 | 1.14 |
| Cleaning | 4.55 | 1.30 |
| Train | 3.49 | 1.31 |
| Cooking | 3.95 | 1.37 |
| Restaurant | 2.81 | 1.24 |
| Karaoke | 3.91 | 1.67 |
| Preparation | 3.53 | 1.22 |
| Internet shopping | 3.21 | 1.21 |
| Hairdresser | 3.70 | 1.32 |
| Hospital | 5.16 | 1.25 |
| Bath | 3.13 | 1.23 |
| PC | 3.42 | 1.23 |
| Movie | 3.18 | 1.36 |
| Driving | 3.65 | 1.50 |
| Pet | 3.87 | 1.51 |
